# T-Bet independent development of IFNγ secreting natural T helper 1 cell population in the absence of Itk

**DOI:** 10.1038/srep45935

**Published:** 2017-04-13

**Authors:** Arun K. Kannan, Sonia Mohinta, Weishan Huang, Lu Huang, Nicholas Koylass, Judith A. Appleton, Avery August

**Affiliations:** 1 Department of Microbiology & Immunology, Cornell University, Ithaca, NY, 14853, USA; 2 Baker Institute for Animal Health, Cornell University, Ithaca, NY, 14853, USA.

## Abstract

Th1, Th2, Th9 and Th17 cells are conventional CD4^+^ effector T cells identified as secretors of prototypical cytokines IFNγ, IL4, IL9, and IL-17A respectively. Recently, populations of natural Th17 and Th1 cells (nTh17 and nTh1) with innate-like phenotype have been identified in the thymus that are distinct from conventional Th17 and Th1 cells. The absence of the Tec family kinase Interleukin-2 inducible T cell kinase (Itk) results in T cell immunodeficiency in mice and humans. Here we show that Itk negatively regulates the development of nTh1 cells that express IFNγ in a Tbet independent manner, and whose expansion can be enhanced by IL4. Furthermore, we show that robust induction of IL4 responses during *Trichinella spiralis* infection enhance the presence of nTh1 cells. We conclude T cell receptor signaling via Itk controls the development of natural Th1 cells, which are expanded by the presence of IL4.

Upon encountering an antigen, a naïve CD4^+^ T cell differentiates into distinct effector T helper cell lineages. These subsets are distinguished by the expression of lineage specific transcription factor, their cytokine profile and effector function. In response to antigen from an intracellular pathogen such as a virus, T cells differentiate to a Th1 subtype by upregulating its master transcription factor Tbet and secrete IFNγ. In presence of extracellular pathogen or parasite T cells differentiate to Th2 subtype by upregulating GATA3 and secretion of IL-4, IL-5 and IL-13. Th9 cells express Purine-rich 1 (PU.1) and secrete IL-9, while Th17 cells are generated in response to extracellular bacteria and fungi, express RAR–related Orphan Receptor gamma T (RORγt) and secrete IL-17^1^,^2^,^3^. Aside from these conventional CD4^+^ T cell effectors, a number of T cell populations have been identified that also secrete T-helper cytokines, including those that have innate effector function such Invariant Natural Killer T cells (*i*NKT) cells and γδ T cells. Unlike conventional T cells, these innate T cells have a limited TCR repertoire, show features of activated cells and are primed for rapid effector cytokine secretion even during their thymic development^4^,^5^. Additionally, a population of Th17 (nTh17) cells with innate-like phenotype has recently been identified in the thymus that is distinct from conventional Th17 cells. These natural Th17 cells have a skewed T cell Receptor (TCR) arrangement and have a differential requirement for thymic selection, distinct from their conventional counterpart^6^,^7^,^8^,^9^,^10^. These nTh17 cells have been shown to be generated in response to both self as well as non-self antigens and play a role in mediating host immune responses. In genetically manipulated mice with impaired TCR signaling, including those lacking the Tec kinase Itk, we and others have reported the emergence of CD8^+^ T cells with innate and memory cell-like characteristics, dependent on enhanced IL-4 signaling^11^,^12^,^13^,^14^,^15^,^16^,^17^,^18^. More recently, a population of innate memory like CD4^+^ T cells (nTh1) has been identified in the thymus with characteristics of Th1 cells. This population is generated in Class II Major Histocompatibility Complex Transactivator transgenic (CIITAtg) and BALB/c mice in the context of availability of increased IL-4^19^,^20^. These “nTh1” cells share features of innate memory CD8^+^ T cells including expression of activation markers, the transcription factor Eomesodermin (Eomes) and rapid secretion of Th1-like cytokines upon stimulation.

The Tec family kinase Interleukin-2 inducible T cell kinase (Itk) is critical for optimal signaling through the TCR and in its absence, humans and mice exhibit T cell immunodeficiency. Itk has been shown to differentially regulate the development and effector function of conventional as well as non-conventional innate-like T cells^21^,^22^,^23^,^24^. In conventional T cells, we and other have shown that the absence of Itk results in severely impaired Th2 and Th17 responses, which is attributed to impaired activation of key signaling molecules downstream of the TCR including reduced nuclear translocation of NFAT. By contrast we and others have shown that the absence of Itk enhances the development of T regulatory cells^25^,^26^. These findings clearly support a role for Itk in regulating pathways that control development of effector Th cells and their cytokine production. Here we identify and characterize a novel population of IFNγ expressing CD4^+^ T cells that are expanded in the absence of Itk. We show that these nTh1 cells are generated from a subset of T cells that receive lower TCR signals in the thymus and have skewed TCR repertoire with a preferential Vβ3 usage. More importantly, we show that the development of this population is independent of the Th1 lineage master transcription factor T-bet, and can develop in the absence IL-4 signaling, although IL-4 can drive both the expansion and expression of Eomes by this population. In line with this, we show that there is an expanded population of nTh1 cells in mice infected with *Trichinella spiralis*, a parasite that induces a strong Th2 response later on during the immune response. We suggest that these are natural Th1 (nTh1) cells analogous to the recently reported innate Eomes+ CD4^+^ T cells generated in CIITAtg and BALB/c mice. These results suggest that nTh1 cells are generated under physiological conditions of lower TCR signaling and can be expanded by IL4 signaling induced by natural infection *in vivo*.

## Materials and Methods

### Mice

WT, T-bet^−/−^ and IFNγ^−/−^ mice were purchased from Jackson Laboratory. IL13^−/−^ (gift of Dr. Margaret Bynoe, Cornell University), IL4Rα^−/−^ (gift of Dr. Frank Brombacher, University of Cape Town, South Africa, via Dr. Fred Finkelman (University of Cincinnati, OH, USA), T-bet^−/−^, IFNγ^−/−^, IL4Rα^−/−^ and IL13^−/−^ mice were backcrossed to Itk^−/−^ mice for at least five generations to generate respective double knockout mice. All mice were on the C57BL/6 background, were used between 6–12 weeks of age, and both males and females mice were used. Animals were housed in specific pathogen free facilities at Cornell University and all experiments were approved by the Office of Research Protection’s Institutional Animal Care and Use Committee at the Cornell University. All experimental methods were performed in accordance with the relevant guidelines and regulations at Cornell University.

### *Ex vivo* stimulation and flow cytometry

Freshly isolated thymocytes or splenocytes were stimulated with 50 ng/ml of PMA (Sigma) and 1 μg/ml of Ionomycin (Sigma) in the presence of 1–5 μg/ml of Brefeldin A (Sigma) for 4–5 hours. Stimulated cells were stained for the indicated surface markers antibodies against CD4 (clone # GK1.5), CD8 (clone #53-6.7), TCRβ (clone # H57-597), CD44 (clone IM7), alpha GalCer (NIAID Tetramer Facility), NK1.1 (clone PK136), IFNγ (clone XMG1.2), CD69 (clone H1.2F3), CD24 (clone M1/69), CD5 (clone 53-7.3), Nur77 (clone 12.14), Vβ3 (clone 8F10), Eomesodermin (clone Dan11mag), and subsequently fixed and permeabilized using the Foxp3 fixation/permeabilization kit according to manufacturers’ instructions and stained for the indicated intracellular proteins. Data was acquired on a LSR II (BD Biosciences) and analyzed using FlowJo software (Tree Star).

### Fetal Thymic Organ Cultures (FTOCs)

FTOCs were performed as described previously^27^. Briefly, fetal thymic lobes were isolated from embryonic day 16.5 embryos and cultured on inserts in a 0.4 μm 6-well transwell plate (Costar) with 1.5 ml of RPMI medium in the lower chamber. The medium was changed on the 4^th^ day of culture and the single cell suspensions of the thymic lobes were obtained after 8 days in culture.

### T. spiralis Infection

*T. spiralis* first-stage larvae (L_1_) was isolated from infected rats as previously described^28^. For infection of mice, 300 L_1_ larvae in 2% nutrient broth (Difco)−0.6% gelatin (Fischer Scientific) were administered by oral gavage. Thymocytes were isolated from mice euthanized at the indicated days post infection.

### Statistical analysis

Student’s *t* test and ANOVA were performed using Prism software to evaluate statistical significance between samples sets or multiple groups, which had similar variance, with *p* < 0.05 considered statistically significant. For animal studies, power analysis and prior experience in performing similar studies were used to determine sample sizes. Since each experimental group represents a distinct genetically engineered mouse strain or group of mice treated the same way and analyzed at different time points (e,g, *T. Spiralis* experiments), mice were not randomized nor were the investigators blinded in these experiments.

## Results

### Absence of Itk enhances development of natural Th1 cells

**Figure 1:**
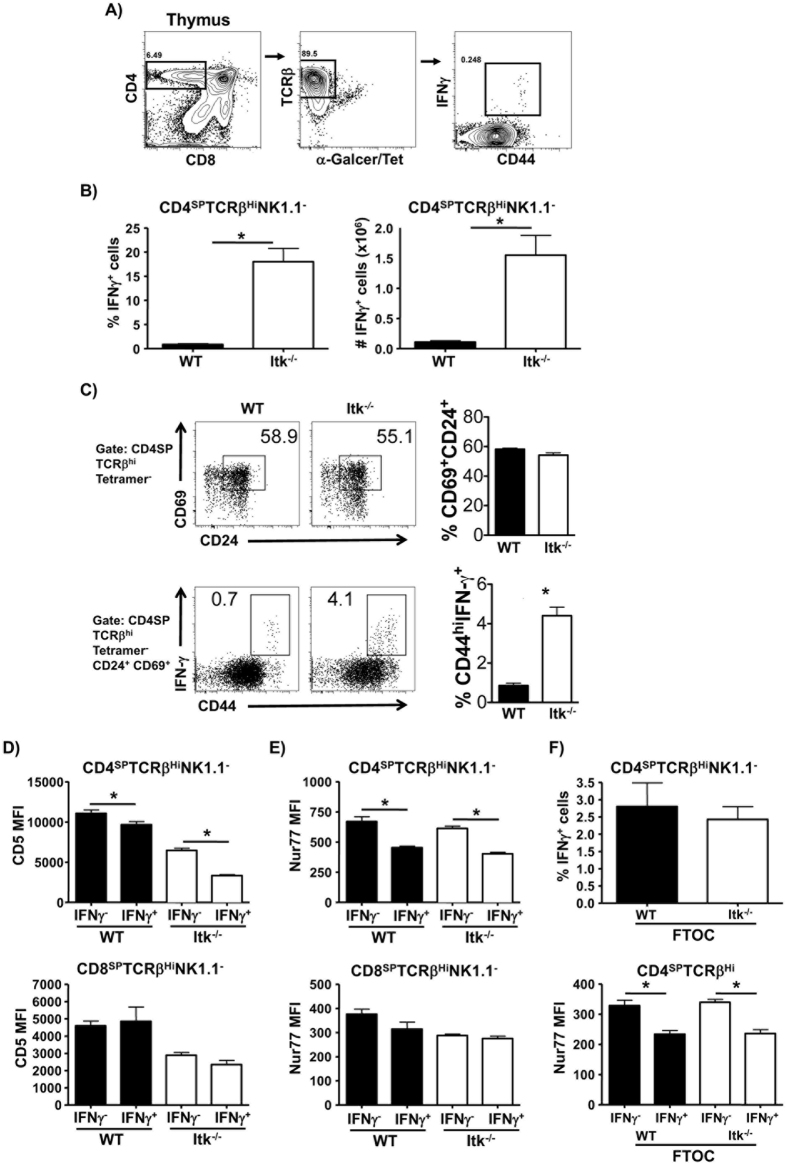
Itk signaling negatively regulates the development of nTh1 cells. (**A**) Gating strategy for the analysis of nTh1 cells. Thymocytes from WT and Itk^−/−^ mice were stimulated with PMA and Ionomycin (P/I) in the presence of Brefeldin A and CD4SPTCRβ^high^αGalCer Tetramer^−^ (or NK1.1^−^ in some experiments) cells were analyzed for the expression of IFNγ by FACS. (**B**) Thymocytes from WT and Itk^−/−^ mice were stimulated with PMA and Ionomycin (P/I) in the presence of Brefeldin A and CD4SPTCRβ^high^NK1.1^−^ cells were analyzed for the expression of IFNγ by FACS. Plotted as proportion (left panel) and number (right panel) of nTh1 cells in the thymus (n = 8 (WT)/ n=10 (Itk^−/−^)). (**C**) Thymocytes from WT and Itk^−/−^ mice were stimulated with PMA and Ionomycin (P/I) in the presence of Brefeldin A and CD4SPTCRβ^high^αGalCer Tetramer^−^ cells were analyzed for CD69 and CD24 as indicated, followed by the expression of IFNγ by FACS (n = 3/group). (D-F) Thymocytes from WT or Itk^−/−^ mice treated as in (**B**) was analyzed for the expression of CD5 (n=12 (WT)/ n=7 (Itk^−/−^)) (**D**) and Nur77 (n=4 (WT)/ n=4 (Itk^−/−^)) (**E**) by CD4SPTCRβ^high^NK1.1^−^ cells (top panel) and CD8SPTCRβ^high^NK1.1^−^ cells (bottom panel) (n = 7–12/group). (F) Cells isolated from D8 FTOCs generated from WT and Itk^−/−^ embryos (embryonic day 16.5) were treated as in (**B**) and analyzed for the expression of IFNγ (top panel), Nur77 (bottom panel) using FACS (3 independent cell preps of FTOCs with 2–4 fetal thymic lobes per cell prep). Data is cumulative of at least 2 independent experiments. Error bars represent mean ± SEM, *p < 0.05 calculated by unpaired Students’ t test.

We have previously shown that naïve peripheral CD4^+^ T cells in Itk^−/−^ mice carry preformed mRNA for IFNγ and the Th1 transcription factor T-bet, and rapidly produce IFNγ upon stimulation^27^. We also previously showed that elevated T-bet was a function of the preexisting IFNγ expression in these cells, and that this “primed” nature of naïve Itk^−/−^ CD4^+^ T cells resulted in enhanced preferential Th1 differentiation *in vitro*^27^,^29^. To determine if this was a characteristic that these cells acquired during development in the thymus, we analyzed expression of IFNγ and T-bet in CD4 single positive (SP) thymocytes following stimulation with PMA and Ionomycin, gating out α-Galactosyl Ceramide/CD1d (α-GalCer/CD1d) tetramer positive (or in some cases NK1.1 positive) *i*NKT cells which can rapidly produce this cytokine (see Fig. 1A for gating strategy). We found that a percentage of both WT and Itk^−/−^ CD4SPTCRβ^high^NK1.1^−^ cells rapidly produced IFNγ upon stimulation, but that there was a significantly higher proportion and number of Itk^−/−^ CD4SP cells that produced IFNγ (Fig. 1B). Thus these cells are similar to the recently described nTh1 cells, in that they develop in the thymus, and can rapidly produce cytokine, in this case IFNγ, upon stimulation^19^.

It is possible that these IFNγ producing cells are not actually thymocytes, but mature peripheral T cells that have recirculated back into the thymus. To exclude such cells, we used markers to identify mature CD4SP thymocytes that have not yet left the thymus as reported by Fink and colleagues^30^, and found that the nTh1 cells were still found in the thymus, and that their proportions were elevated in the absence of Itk (Fig. 1C).

### nTh1 cells receive lower TCR signals during development

The strength of signaling through the TCR has been shown to regulate a number of naturally occurring effector populations in the thymus including natural T regulatory cells (nTregs), γδ T cells and *i*NKT cells^5^. Since the absence of Itk would be predicted to result in lower TCR signal strength we determined if the development of nTh1 cells is regulated by TCR signal strength by analyzing the expression of CD5, a marker of TCR signal strength in T cells^31^. Consistent with the notion that T cells with lower TCR signal strength develop into nTh1 cells, we found that the nTh1 population (IFNγ^+^) in both WT and Itk^−/−^ thymus had significantly lower expression of CD5 compared to the IFNγ^−^ non-nTh1 population (Fig. 1D, top panel). This is further supported by the fact that WT and Itk^−/−^ nTh1 cells also have lower expression for Nur-related factor 77 (Nur77), another surrogate marker for TCR signal strength (Fig. 1E, top panel)^32^. Interestingly, we find no difference in the level of expression of CD5 or NuR77 in the CD8SPTCRβ^high^NK1.1^−^IFNγ^+^ population compared to CD8SPTCRβ^high^NK1.1^−^IFNγ^−^ population in both WT and Itk^−/−^ mice (Fig. 1D,E, bottom panels), further supporting differences in development of these nTh1 cells. Note again that Itk^−/−^ CD4SP as well as CD8SP cells also had lower expression of CD5 and Nur77 compared to their WT counterparts, which is in line with previous reports that there is reduced TCR signaling in the absence of Itk^33^. To further confirm the ontogeny of these cells we performed fetal thymic organ cultures (FTOC) of fetal thymic lobes isolated from WT and Itk^−/−^ embryos (embryonic day 16.5). FTOCs analyzed at 8 days’ post culture, revealed a significant proportion of these IFNγ^+^ cells with lower Nur77 expression within the CD4SP compartment (Fig. 1F), suggesting that these cells were derived from the thymus and did not represent a population that had recirculated into the thymus from the periphery. These data suggest that T cells with lower affinity or avidity TCR that translates to lower signal strength preferentially differentiate into nTh1 cells.

### nTh1 cells preferentially express Vβ3 T cell receptor

**Figure 2:**
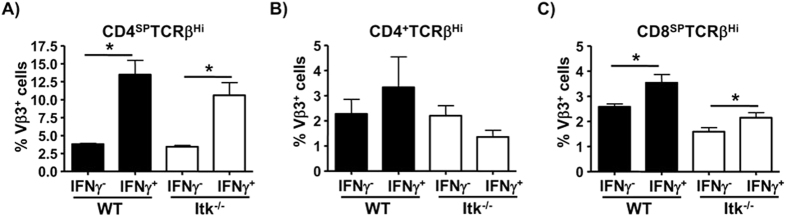
nTh1 cells preferentially express Vβ3 TCR. WT and Itk^−/−^ thymocytes or splenocytes were stimulated with P/I in the presence of Brefeldin A and analyzed. (**A**) CD4SPTCRβ^high^ thymocytes (n=10 (WT)/ n=11 (Itk^−/−^)), (**B**) CD4^+^ TCRβ^+^ splenocytes (n=3 (WT)/ n=4 (Itk^−/−^)) and (C) CD8SPTCRβ^high^ thymocytes were analyzed for the expression of Vβ3 by IFNγ^+^ versus IFNγ^−^ populations using FACS (n=10 (WT)/ n=11 (Itk^−/−^)). Data is cumulative of at least 2 independent experiments. Error bars represent means ± SEM, *P < 0.05 calculated by unpaired Students’ t test.

We next determined whether nTh1 cells used different Vβ in their TcRs. We found that the nTh1 cells that develop in the presence or absence of Itk showed higher enrichment for cell bearing Vβ3 compared to non-nTh1 CD4SP cells (Fig. 2A). This preferential Vβ3 usage was not seen in the Th1 cells that analyzed in the periphery (Fig. 2B), although this could be secondary to the fact that we are potentially analyzing a mixed population of natural and conventional Th1 cells in the periphery. While we did not see any difference in the Vβ3 usage between CD4^+^IFNγ^+^ and CD4^+^IFNγ^−^ populations in the periphery, whether nTh1 cells are the precursors of the CD4^+^Vβ3^+^IFNγ^+^ population we find in the periphery warrants further investigation. Although we saw a slight but significant increase in the enrichment of Vβ3^+^ cells within CD8^SP^TCRβ^high^NK1.1^−^IFNγ^+^ compared to CD8^SP^TCRβ^high^NK1.1^−^IFNγ^−^ population this was significantly lower than the skew we see in CD4^SP^ population (Fig. 2C).

### Role of T-bet in the development of nTh1 cells

**Figure 3:**
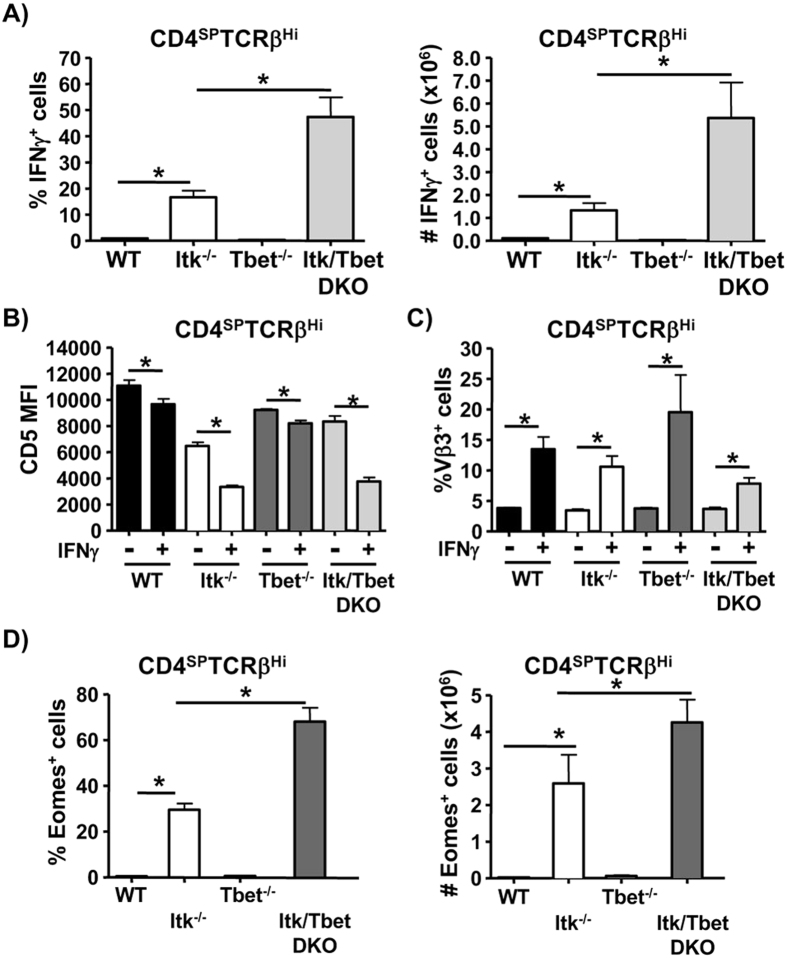
T-bet is dispensable for the development of nTh1 cells. (**A**) Thymocytes from the indicated mice were stimulated with P/I in the presence of Brefeldin A and analyzed for the expression of IFNγ by CD4SP TCRβ^high^ cells and represented as proportion (left panel) or number (right panel) of nTh1 cells (n=11 (WT)/ n=12 (Itk^−/−^)/ n=4 (Tbet^−/−^)/ n=7 (Tbet^−/−^Itk^−/−^)). (**B**,**C**) CD4SP TCRβ^high^ cells treated as in (**A**) were analyzed for the expression of CD5 (n=12 (WT)/ n=7 (Itk^−/−^)/ n=3 (Tbet^−/−^)/ n=5 (Tbet^−/−^Itk^−/−^)) (**B**) and Vβ3 TCR (n=9 (WT)/ n=11 (Itk^−/−^)/ n=3 (Tbet^−/−^)/ n=5 (Tbet^−/−^Itk^−/−^)) (**C**) by IFNγ^+^ versus IFNγ^−^. (**D**) CD4SP TCRβ^high^ cells (n=11 (WT)/ n=12 (Itk^−/−^)/ n=4 (Tbet^−/−^)/ n=7 (Tbet^−/−^Itk^−/−^)) treated as in (**A**) were analyzed for the expression of Eomes by FACS and represented as proportion (left panel) or number (right panel) of Eomes^+^ cells (n = 4–12/group). Data is cumulative of at least 2 independent experiments. Error bars represent means ± SEM, *P < 0.05 calculated by unpaired Students’ t test.

It is well established that the T-box transcription factor T-bet is important for the differentiation and function of Th1 cells^34^, so we examined mice lacking T-bet or T-bet and Itk for thymocytes with the properties of nTh1 cells. We found that T-bet was not essential for the generation of nTh1 cells as Itk/T-bet DKO mice had an even higher proportion and number of nTh1 cells in the CD4SP cell compartment than Itk^−/−^ mice (Fig. 3A). Similar to the nTh1 cells that develop in the WT and Itk^−/−^ mice, the cells that develop in the T-bet^−/−^ and Itk/T-bet DKO mice had lower expression of CD5 and were enriched for Vβ3^+^ cells (Fig. 3B,C). These results suggest that T-bet was not only dispensable for the development nTh1 cells but the absence of T-bet on the Itk^−/−^ background promoted the development and/or expansion of nTh1 cells.

Eomesodermin (Eomes) is another T-box transcription factor that has been shown to be able to regulate the production of IFNγ^35^, and so we next determined the expression of Eomes in nTh1. We found that while WT and Tbet^−/−^ nTh1 cells rarely expressed Eomes, this factor was highly upregulated in the nTh1 cells from Itk^−/−^ and Itk/T-bet DKO mice (Fig. 3D).

### IL4 signaling expands nTh1 cells and induces their expression of Eomes, but is not required for their development

**Figure 4:**
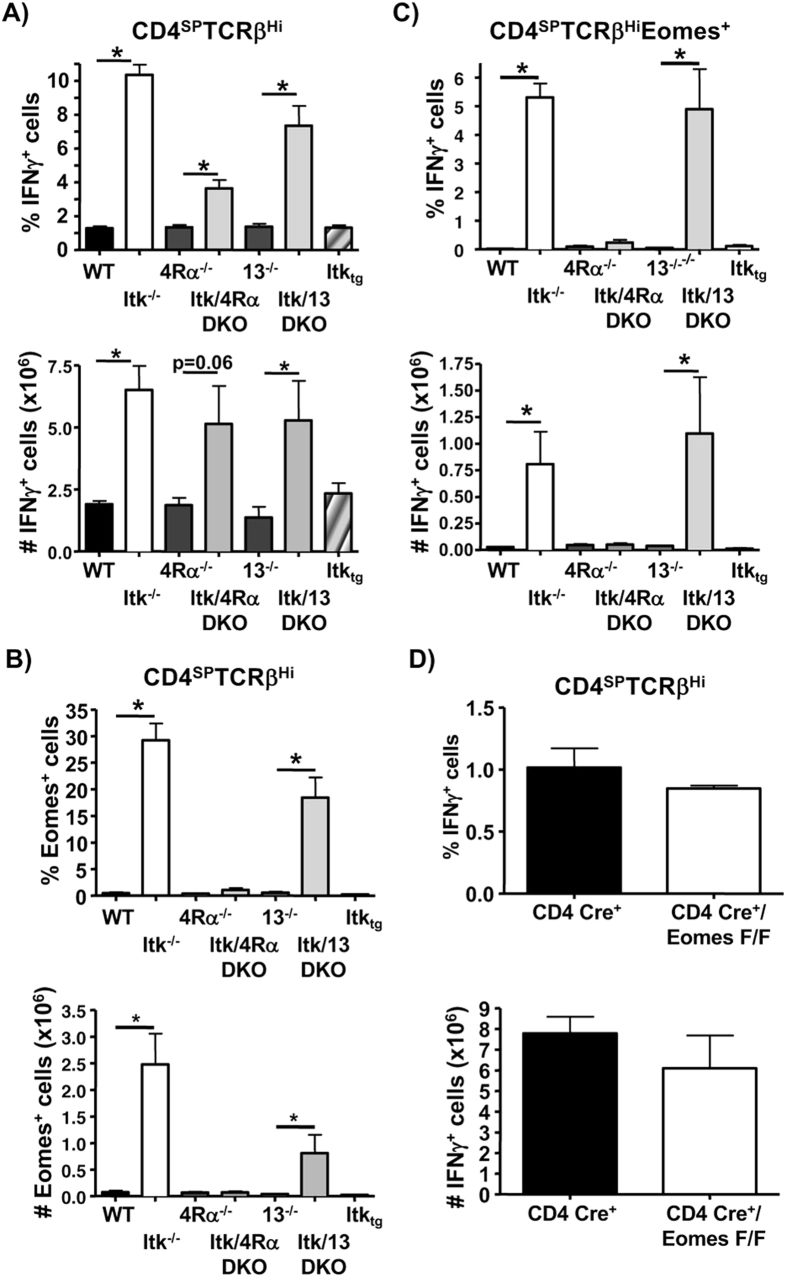
IL4 signaling expands the nTh1 population but is dispensable for their development. (**A**,**B**) Thymocytes from WT (n=9), Itk^−/−^ (n=12), IL4Rα^−/−^ (n=8), Itk^−/−^/IL4Rα^−/−^ (n=9), IL13^−/−^ (n=6) and Itk^−/−^/IL13^−/−^ (n=8) were stimulated and analyzed for the expression of IFNγ (**A**), Eomes (**B**) and IFNγ/Eomes double positive (**C**) cells in the CD4SP TCRβ^high^ population. Data presented as proportion (left panel) or number (right panel) of cells (n = 3–12/group). (**D**) Thymocytes from CD4-Cre^+^ and CD4-Cre/Eomes^F/F^ mice were stimulated and analyzed for the expression of IFNγ by CD4SP TCRβ^high^ cells and represented as proportion (top panel) or number (bottom panel) of cells (n = 3/group). Data is cumulative of at least 2 independent experiments. Error bars represent means ± SEM, *p < 0.05 calculated by unpaired Students’ t test.

We and others have recently shown that IL4 signaling is critical for the development of CD8 innate memory phenotype (IMP) T cells that develop in the absence of Itk^11^,^12^,^36^. These CD8 IMP cells have preformed mRNA for IFNγ and rapidly produce this cytokine upon stimulation, similar to what we see for the nTh1 cells^15^,^37^,^38^. We therefore wanted to determine if IL4 pathway was involved in the development of these cells. We found that although Itk/IL4Rα DKO mice had a lower proportion of nTh1 cells compared to Itk^−/−^ controls, these mice had a higher proportion of nTh1 cells compared to IL4Rα^−/−^ controls. Analysis of the related Th2 cytokine IL13 (using Itk/IL13 DKO mice) revealed similar findings suggesting that IL-13 may also contribute (Fig. 4A). These results suggest that while the IL4 signaling axis may promote the expansion of nTh1 cells, unlike CD8 IMP T cells, it is not a requirement for the development of these cells^12^. However, the IL4 signaling axis is responsible for the expression of the Eomes transcription factor in nTh1 cells, as in the absence of the IL4Rα, there was a complete loss of expression of IFNγ^+^/Eomes^+^ nTh1 cells (as well as Eomes^+^ cells) in Itk^−/−^ mice, but not in the absence of IL13 (Fig. 4B,C). However, analysis of thymocytes from T cell specific (CD4-Cre) Eomes mice revealed no difference in nTh1 cells compared to control mice (Fig. 4D). Note that re-expression of Itk on the Itk^−/−^ background in T cells reverts the proportion of nTh1 cells to WT levels (Fig. 4A–C)^15^. These results suggest that while IL4 signaling is dispensable for the development of nTh1 cells, it is essential for the induction of Eomes expression in these cells.

### Expansion of nTh1 cells under robust Th2 conditions

**Figure 5:**
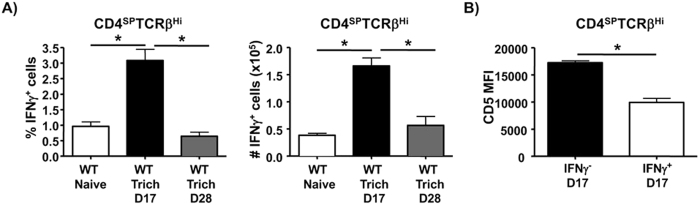
Increase in the expression of Th2 cytokines *in vivo* during infection with *T. spiralis* can promote the expansion of nTh1 cells. (**A**) Thymocytes isolated from uninfected (n=6), day 17 (n=12) and day 28 (n=4) *T. spiralis* infected WT mice were stimulated as in Fig. 1 and analyzed for the expression of IFNγ by CD4SP TCRβ^high^ cells and plotted as proportion (left panel) or number (right panel) of nTh1 cells. (**B**) Thymocytes from mice infected as in (**A**) were analyzed for the expression of CD5 by IFNγ^+^ and IFNγ^−^ CD4SP TCRβ^high^ cells (n = 8/group). Data is cumulative of at least 2 independent experiments. Error bars represent means ± SEM, *p < 0.05 calculated by unpaired Students’ t test.

The results so far suggest that T cells with lower affinity TCR preferentially differentiate into nTh1 cells in the thymus, a process that can be augmented by the loss of Itk which dampens TCR signaling, and that the expansion of these cells can be promoted by IL4 signaling. However, it is unclear if this represents a physiological pathway for the generation and/or expansion of nTh1 cells. To test this, we analyzed the thymus of WT mice under conditions of an infection that generates a strong Th2 response. Infection with *Trichinella spiralis (T. spiralis*) induces a strong Th2-mediated immunity that peaks around 17–22 days post infection (dpi) and subsides by 28 dpi^39^. We found that infection with *T. spiralis* led to a marked increase in the proportion and number of nTh1 cells in the thymus that was coincident (17 dpi) with a robust Th2 response, with lower level expression of CD5 (Fig. 5A,B). The proportion and number of these nTh1 cells was back to basal levels by 28 dpi when the Th2 response had subsided. These results suggest that physiological signals that result in strong production of IL4 such as infection with the parasite *T. spiralis*, can induce the expansion of nTh1 cells in the thymus of WT mice, providing further evidence for the idea that nTh1 cells develop from low affinity TCR interactions in the thymus under normal conditions, and are expanded in the presence of IL4.

## Discussion

While conventional T helper cells such as Th1 cells require differentiation in the presence of specific cytokine conditions over a period of days to take on full effector function, the recently described natural Th1 cells have develop in the thymus, have preformed mRNA for IFNγ, and can rapidly produce IFNγ upon stimulation^19^,^20^. Here we show that development of nTh1 cells is enhanced in the face of reduced signaling as a consequence of the absence of the kinase Itk. We also show that surprisingly, enhanced development of nTh1 cells was independent of the Th1 master transcription factor T-bet. We also show that while the Th2 cytokine is not required for the development of nTh1 cells, the presence of IL4 signal allows the expansion of these cells. Our findings are similar to what has been reported by Berg and colleagues, although they did not refer to this population as nTh1 cells^40^. Our data suggest a new function for Itk, and suggest that patients with mutations in this kinase may have enhanced Th1 responses due to the presence of nTh1 cells.

Natural Th1 cells were first reported to develop in CIITA transgenic mice, express expression of Eomes and can undergo expansion in the presence of IL4^19^,^20^. These cells were suggested to develop due to low affinity/low avidity interactions with the TcR. Our findings with Itk deficient mice, where we observe enhanced development of these cells support the conclusion that these cells develop in the face of reduced TcR signaling. We note that in the FTOC *in vitro*, we can detect the presence of these cells in both WT and Itk^−/−^ thymus, suggesting that they develop under both conditions, however, we do not see an increase in these cells in these cultures absence of Itk. This may reflect the fact that the *in vitro* conditions are sufficient for the development of these cells, but not their expansion in the absence of Itk. This expansion may be dependent in part on the presence of IL4, or other conditions *in vivo* that is not recapitulated *in vitro*, since we see elevated proportions of these cells in the absence of Itk even when potential recirculated T cells are excluded by flow cytometric gating. Interestingly, the previous report of nTh1 cells did not find evidence for enrichment for the enrichment of particular TcR Vβ usage, although Vβ3 expression was not examined. We show here that the nTh1 cells that develop in WT and Itk^−/−^ mice are enriched for expression of Vβ3, and that these cells show evidence of having received reduced TcR signaling. Thus the absence of Itk may enhance the development of these cell due to the reduced TcR signals that developing thymocytes receive.

Conventional Th1 cells utilize the transcription factor T-bet to enforced differentiation toward the Th1 lineage. We were therefore surprised to find that nTh1 cells develop in the absence of T-bet, and actually Itk/T-bet double knockout mice have even more enhanced development of nTh1 cells. Although there are reports that conventional Th1 cells are not completely dependent on T-bet for their differentiation, T-bet plays a significant role in this process. Natural Th1 cells express the related transcription factor Eomes, although we suggest that Eomes is not required for their development, but is actually induced by the presence of IL4 during the expansion of these cells by this cytokine. This model is supported by the fact that we continue to observe enhanced development of these nTh1 cells in the absence of IL4 signaling, although they lack significant Eomes expression, and their proportion is reduced. However, our data do not exclude the possibility that either Eomes or T-bet is responsible for the development of these cells and a more definitive conclusion will come from analysis of T-bet/Eomes double knockout mice.

We have recently shown that Itk tunes the response of CD8^+^ T cells to IL4, resulting in the development of what we and others have termed innate memory phenotype (IMP) T cells, and we suggest that Itk may perform a similar function in these CD4^+^ nTh1 cells, such that the absence of Itk leads to reduced TcR signals, predisposing the development nTh1 cells, which acquire expression of Eomes and are then expanded by enhanced sensitivity to IL4^12^,^18^. However, CD8^+^ IMP cells are completely abrogated in the absence of IL4 signaling^12^,^18^, unlike the case for these nTh1 cells, which are still present in the absence of IL4 signaling, albeit at reduced proportions. This suggests that IL4 is more important for the expansion of nTh1 cells, as compared to CD8^+^ IMP cells, where IL4 is required for their development. We note that Tofukuji *et al*. have reported on the generation of a novel Th1 population that can be generated by exposure of naïve CD4^+^ T cells to TcR signals in the presence of TGFβ and IL4^41^. Interestingly, these cells also express Eomes. Furthermore a population of Eomes expressing CD4^+^ T cells that are cytotoxic to tumors can be induced by 4-1BB^42^. Further work will be needed to determine whether these cells are related.

Our work also suggests that cells with a similar phenotype expand in the thymus during infection with *T. spiralis*, which drives a robust Th2/IL4 response. This suggests that these cells may contribute to the immune response during robust Th2 responses. In addition, in allergic inflammatory diseases such as allergic asthma, where the levels of IL4 are elevated, it is possible that this could drive the presence of nTh1 cells. However, while this may be possible in children with allergic asthma, where IFN-γ- producing CD4^+^ T cells are frequently observed, although it should be made clear that it is not known whether these cells are allergen-derived, or are derived via a mechanism that involves the influence of IL-4 on developing T cells^43^. While IFN-γ- producing CD4^+^ T cells are also observed in adults with allergic asthma^44^, this mechanism of IL-4 induction of these cells is probably less likely to occur given the thymus is most likely already involuted^45^. However, we speculate that if there is an IL-4 > nTh1 > IFN-γ axis in diseases such as allergic asthma, attempts to reduce IL-4 using monoclonal antibody approaches (such as in pascolizumab^46^), may disrupt a homeostatic network that may have unknown consequences. Regardless, lacking specific markers to deplete these cells, we are unable to directly test their role in infection or in diseases such as allergic asthma at this time. Nevertheless, taken together, this work supports to existence of a novel population of nTh1 cells that develop under conditions of low TcR signal strength as seen in the absence of Itk, and under the influence of IL4. It will be of considerable interest to determine the function of these cells in the immune response.

## Additional Information

**How to cite this article**: Kannan, A. K. *et al*. T-Bet independent development of IFNγ secreting natural T helper 1 cell population in the absence of Itk. *Sci. Rep.* **7**, 45935; doi: 10.1038/srep45935 (2017).

**Publisher's note:** Springer Nature remains neutral with regard to jurisdictional claims in published maps and institutional affiliations.

## References

[b1] Kaplan M. H., Hufford M. M. & Olson M. R. The development and *in vivo* function of T helper 9 cells. Nat Rev Immunol 15, 295–307 (2015). 25848755 10.1038/nri3824 PMC4445728

[b2] Coquet J. M., Rausch L. & Borst J. The importance of co-stimulation in the orchestration of T helper cell differentiation. Immunol Cell Biol 93, 780–788 (2015). 25801480 10.1038/icb.2015.45

[b3] Zhu J., Yamane H. & Paul W. Differentiation of effector CD4 T cell populations. Annu Rev Immunol. 28, 445–489 (2010). 20192806 10.1146/annurev-immunol-030409-101212 PMC3502616

[b4] Macho-Fernandez E. & Brigl M. The Extended Family of CD1d-Restricted NKT Cells: Sifting through a Mixed Bag of TCRs, Antigens, and Functions. Front Immunol 6, 362 (2015). 26284062 10.3389/fimmu.2015.00362 PMC4517383

[b5] Zarin P. & Chen E. L. In, T. S., Anderson, M. K. & Zuniga-Pflucker, J. C. Gamma delta T-cell differentiation and effector function programming, TCR signal strength, when and how much? Cell Immunol 296, 70–75 (2015). 25866401 10.1016/j.cellimm.2015.03.007

[b6] Zuniga L. A., Jain R., Haines C. & Cua D. J. Th17 cell development: from the cradle to the grave. Immunol Rev 252, 78–88 (2013). 23405896 10.1111/imr.12036

[b7] Jenkinson W. E. . Natural Th17 cells are critically regulated by functional medullary thymic microenvironments. J Autoimmun 63, 13–22 (2015). 26143957 10.1016/j.jaut.2015.06.008 PMC4570931

[b8] Conti H. R. . Oral-resident natural Th17 cells and gammadelta T cells control opportunistic Candida albicans infections. J Exp Med 211, 2075–2084 (2014). 25200028 10.1084/jem.20130877 PMC4172215

[b9] Massot B. . TLR-induced cytokines promote effective proinflammatory natural Th17 cell responses. J Immunol 192, 5635–5642 (2014). 24808372 10.4049/jimmunol.1302089

[b10] Tanaka S. . Natural occurring IL-17 producing T cells regulate the initial phase of neutrophil mediated airway responses. J Immunol 183, 7523–7530 (2009). 19890042 10.4049/jimmunol.0803828

[b11] Prince A. L. . Development of innate CD4^+^ and CD8^+^ T cells in Itk-deficient mice is regulated by distinct pathways. J Immunol 193, 688–699 (2014). 24943215 10.4049/jimmunol.1302059 PMC4114307

[b12] Huang W., Huang F., Kannan A. K., Hu J. & August A. ITK tunes IL-4-induced development of innate memory CD8^+^ T cells in a gammadelta T and invariant NKT cell-independent manner. J Leukoc Biol 96, 55–63 (2014). 24620029 10.1189/jlb.1AB0913-484RR PMC4056274

[b13] Huang W., Hu J. & August A. Cutting edge: innate memory CD8^+^ T cells are distinct from homeostatic expanded CD8^+^ T cells and rapidly respond to primary antigenic stimuli. J Immunol 190, 2490–2494 (2013). 23408840 10.4049/jimmunol.1202988 PMC4033301

[b14] Gordon S. . Requirements for eomesodermin and promyelocytic leukemia zinc finger in the development of innate-like CD8^+^ T cells. J Immunol. 186, 4573–4578. Epub 2011 Mar 4577 (2011). 21383242 10.4049/jimmunol.1100037 PMC3085897

[b15] Hu J., Sahu N., Walsh E. & August A. Memory phenotype CD8^+^ T cells with innate function selectively develop in the absence of active Itk. Eur J Immunol. 37, 2892–2899 (2007). 17724684 10.1002/eji.200737311 PMC2770953

[b16] Horai R. . Requirements for selection of conventional and innate T lymphocyte lineages. Immunity. 27, 775–785 (2007). 18031697 10.1016/j.immuni.2007.09.012 PMC2377064

[b17] Berg L. J. Signalling through TEC kinases regulates conventional versus innate CD8(+) T-cell development. Nat Rev Immunol 7, 479–485 (2007). 17479128 10.1038/nri2091

[b18] Huang W. & August A. The signaling symphony: T cell receptor tunes cytokine-mediated T cell differentiation. J Leukoc Biol 97, 477–485 (2015). 25525115 10.1189/jlb.1RI0614-293R PMC4338847

[b19] Kang B. H. . Thymic low affinity/avidity interaction selects natural Th1 cells. J Immunol 194, 5861–5871 (2015). 25972479 10.4049/jimmunol.1401628 PMC4456632

[b20] Park S. H. Natural Th1 cells: escape from neglect. Oncotarget 6, 21795–21796 (2015). 26392409 10.18632/oncotarget.5489 PMC4673124

[b21] August A. & Ragin M. J. Regulation of T-cell responses and disease by tec kinase Itk. Int Rev Immunol 31, 155–165 (2012). 22449075 10.3109/08830185.2012.668981

[b22] Gomez-Rodriguez J., Kraus Z. & Schwartzberg P. Tec family kinases in T lymphocytes: Cross-regulation of cytokine production and T cell fates. FEBS Lett 278, 1980–1989 (2011). 10.1111/j.1742-4658.2011.08072.x PMC3117960 21362139

[b23] Andreotti A. H., Schwartzberg P. L., Joseph R. E. & Berg L. J. T-cell signaling regulated by the Tec family kinase, Itk. Cold Spring Harb Perspect Biol 2, a002287 (2010). 20519342 10.1101/cshperspect.a002287 PMC2890196

[b24] Qi Q., Kannan A. K. & August A. Tec family kinases: Itk signaling and the development of NKT alphabeta and gammadelta T cells. FEBS J 278, 1970–1979 (2011). 21362141 10.1111/j.1742-4658.2011.08074.x

[b25] Huang W., Jeong A. R., Kannan A. K., Huang L. & August A. IL-2-inducible T cell kinase tunes T regulatory cell development and is required for suppressive function. J Immunol 193, 2267–2272 (2014). 25063868 10.4049/jimmunol.1400968 PMC4352551

[b26] Gomez-Rodriguez J. . Itk-mediated integration of T cell receptor and cytokine signaling regulates the balance between Th17 and regulatory T cells. J Exp Med 211, 529–543 (2014). 24534190 10.1084/jem.20131459 PMC3949578

[b27] Hu J. & August A. Naive and innate memory phenotype CD4^+^ T cells have different requirements for active Itk for their development. J Immunol. 180, 6544–6552 (2008). 18453573 10.4049/jimmunol.180.10.6544 PMC2836934

[b28] Huang L. . Eosinophils mediate protective immunity against secondary nematode infection. J Immunol 194, 283–290 (2015). 25429065 10.4049/jimmunol.1402219 PMC4272919

[b29] Kannan A., Sahu N., Mohanan S. S. M. & August A. Itk modulates allergic airway inflammation by suppressing IFNγ in naïve CD4^+^ T-cells. J. Allergy Clin. Immunol. 132, 811-820.e811-815 (2013). 10.1016/j.jaci.2013.04.033 PMC4033298 23768572

[b30] Boursalian T. E., Golob J., Soper D. M., Cooper C. J. & Fink P. J. Continued maturation of thymic emigrants in the periphery. Nat Immunol 5, 418–425 (2004). 14991052 10.1038/ni1049

[b31] Kassiotis G., Zamoyska R. & Stockinger B. Involvement of avidity for major histocompatibility complex in homeostasis of naive and memory T cells. J Exp Med 197, 1007–1016 (2003). 12707300 10.1084/jem.20021812 PMC2193871

[b32] Moran A. E. . T cell receptor signal strength in Treg and *i*NKT cell development demonstrated by a novel fluorescent reporter mouse. J Exp Med 208, 1279–1289 (2011). 21606508 10.1084/jem.20110308 PMC3173240

[b33] Berg L. J. Strength of T cell receptor signaling strikes again. Immunity 31, 529–531 (2009). 19833081 10.1016/j.immuni.2009.09.008

[b34] Szabo S. . A novel transcription factor, T-bet, directs Th1 lineage commitment. Cell. 100, 655–669 (2000). 10761931 10.1016/s0092-8674(00)80702-3

[b35] Pearce E. . Control of effector CD8^+^ T cell function by the transcription factor Eomesodermin. Science. 302, 1041–1043 (2003). 14605368 10.1126/science.1090148

[b36] Weinreich M., Odumade O., Jameson S. & Hogquist K. T cells expressing the transcription factor PLZF regulate the development of memory-like CD8^+^ T cells. Nat Immunol. 11, 709–716. Epub 2010 Jul 2014 (2010). 20601952 10.1038/ni.1898 PMC3051359

[b37] Broussard C. . Altered development of CD8^+^ T cell lineages in mice deficient for the tec kinases Itk and Rlk. Immunity. 25, 93–104 (2006). 16860760 10.1016/j.immuni.2006.05.011

[b38] Atherly L. . The Tec family tyrosine kinases Itk and Rlk regulate the development of conventional CD8^+^ T cells. Immunity 25, 79–91 (2006). 16860759 10.1016/j.immuni.2006.05.012

[b39] Gebreselassie N. G. . Eosinophils preserve parasitic nematode larvae by regulating local immunity. J Immunol 188, 417–425 (2012). 22131328 10.4049/jimmunol.1101980 PMC3244516

[b40] Prince A. L. . Innate PLZF^+^CD4^+^ alphabeta T cells develop and expand in the absence of Itk. J Immunol 193, 673–687 (2014). 24928994 10.4049/jimmunol.1302058 PMC4083617

[b41] Tofukuji S. . Identification of a new pathway for Th1 cell development induced by cooperative stimulation with IL-4 and TGF-beta. J Immunol 188, 4846–4857 (2012). 22504655 10.4049/jimmunol.1103799

[b42] Curran M. A. . Systemic 4-1BB activation induces a novel T cell phenotype driven by high expression of Eomesodermin. J Exp Med 210, 743–755 (2013). 23547098 10.1084/jem.20121190 PMC3620352

[b43] Kim J. H. . Different IL-5 and IFN-gamma production from peripheral blood T-cell subsets in atopic and nonatopic asthmatic children. J of Asthma 41, 869–876 (2004). 15641637 10.1081/jas-200038441

[b44] Cho S. H., Stanciu L. A., Holgate S. T. & Johnston S. L. Increased interleukin-4, interleukin-5, and interferon-gamma in airway CD4^+^ and CD8^+^ T cells in atopic asthma. Am J Respir Crit Care Med 171, 224–230 (2005). 15502111 10.1164/rccm.200310-1416OC

[b45] Simpson J. G., Gray E. S. & Beck J. S. Age involution in the normal human adult thymus. Clin Exp Immunol 19, 261–265 (1975). 1212800 PMC1538100

[b46] Hart T. K. . Preclinical efficacy and safety of pascolizumab (SB 240683): a humanized anti-interleukin-4 antibody with therapeutic potential in asthma. Clin Exp Immunol 130, 93–100 (2002). 12296858 10.1046/j.1365-2249.2002.01973.x PMC1906490

